# Colotomy

**Published:** 1890-04-05

**Authors:** 


### SURGERY.

### COLOTOMY.

"Colotomy at Seventy-three Years of Age; Patient Surviving Three Years Later" is the heading of an article in the *Lancet* a short time ago by Mr. George A. Hawkins-Ambler. The operation was performed in consequence of cancerous growth, which could be felt constricting the rectum. Left lumbar colotomy was therefore decided upon. The gut was easily found, stitched to the margin of the wound, and then opened at once. After the operation there was the discharge of long-retained fæces, which exhausted the patient, who required stimulants and opium. Some hard clay-like fæces were also scooped out of the gaping wound. Two months after the operation the patient was sitting up in bed, and in a few weeks afterwards pursued her avocation as a tailoress, being able to walk four miles. As to the cancerous growth, that makes slow progress, but the patient is now losing ground, developing cachexia, and growing less active. Mr. Hawkins-Ambler observes that there was nothing remarkable as regarded the operation, excepting the circumstances under which it was performed. The operating-room was in a small and ill-lighted cottage, the patient at an advanced time of life, and her nurses two daughters completely uninstructed in the art. The case is one which may encourage surgeons to operate under the unfavourable conditions imposed upon them by country life, and the exigencies of practice among the working classes. The immense gain to the patient is perceptible at a glance. In the Retrospect for December 28th last we gave an epitome of Mr. Bryant's "Bradshaw lecture" on colotomy, and it was then remarked that this surgeon has performed colotomy in more than 170 cases, eight surviving the operation from two to six years. Mr. Bryant considers the operation is called for as soon as symptoms of impassable blockage appear, and states that a cancerous malady makes slower progress when the irritation caused by the fæces is removed. Mr. Hawkins-Ambler's case fully supports this observation. Colotomy is not now to be regarded as a *dernier resort* for the relief of chronic intestinal obstruction, for there is sufficient recorded experience to show that it should be had recourse to much more early than has been the practice of the majority of surgeons.

